# The psychological impact of COVID-19 pandemic lockdowns: a review and meta-analysis of longitudinal studies and natural experiments

**DOI:** 10.1017/S0033291721000015

**Published:** 2021-01-13

**Authors:** Gabriele Prati, Anthony D. Mancini

**Affiliations:** 1 Department of Psychology, University of Bologna, Italy, Piazza Aldo Moro, 90, 47521 Cesena (FC), Italy; 2 Department of Psychology, Pace University, Marks Hall, Rm 33, 861 Bedford Road, Pleasantville, NY 10570, USA

**Keywords:** COVID-19, lockdown, mental health, quarantine, SARS-CoV-2, well-being

## Abstract

Lockdowns to control the spread of the coronavirus disease 2019 (COVID-19) have had profound effects on everyday life worldwide, but their effect on mental health remains unclear because available meta-analyses and reviews rely mostly on cross-sectional studies. We conducted a rapid review and meta-analysis of longitudinal studies and natural experiments investigating the relationship between COVID-19 lockdowns and mental health. A total of 25 studies involving 72 004 participants and 58 effect sizes were analyzed. Using a random effects model, we found that lockdowns had small effects on mental health symptoms, *g* = 0.17, s.e. = 0.05, 95% CI (0.06–0.24), *p* = 0.001, but the effects on positive psychological functioning, *g* = −0.12, s.e. = 0.11, 95% CI (−0.33 to 0.09), *p* = 0.27, were not significant. Multivariate analysis of effect sizes revealed significant and relatively small effect sizes for anxiety and depression, while those for social support, loneliness, general distress, negative affect, and suicide risk were not significant. The results indicated substantial heterogeneity among studies, but meta-regression analyses found no significant moderation effects for mean age, gender, continent, COVID-19 death rate, days of lockdown, publication status or study design. The psychological impact of COVID-19 lockdowns is small in magnitude and highly heterogeneous, suggesting that lockdowns do not have uniformly detrimental effects on mental health and that most people are psychologically resilient to their effects.

The spread of the severe acute respiratory syndrome coronavirus 2 (SARS-CoV-2) has resulted in an unprecedented series of lockdowns worldwide. Although these lockdowns have varied in stringency between and within countries, they have substantially altered people's daily lives globally, affecting their work, leisure activities, livelihood, and capacity for in-person social interaction. It is difficult to draw definitive conclusions regarding the impact of coronavirus disease 2019 (COVID-19) lockdowns from prior research, because the COVID-19 lockdowns have marked qualitative differences from those of previous pandemics (e.g. the substantial socio-economic impact, greater degree of stringency, and the variable nature of enforcement). Although one prior systematic review that suggests that general coronavirus infections have minimal effects on symptoms of mental illness (Rogers et al., 2020), this prior review does not address the broader psychological impact of the pandemic lockdowns on general population samples.

In the present review and meta-analysis, we sought to focus on the emerging literature on COVID-19 lockdowns to investigate the psychological impact of lockdown on the general population. Specifically, we reviewed and meta-analyzed studies that included between-group or within-group controls, allowing for clearer inferences regarding the impact of lockdown on mental health. Following previous research (e.g. Haider et al., 2020), we defined lockdown as an emergency and temporary measure imposed by governmental authorities that (1) applies to a city, region, or nation to prevent the spread of the COVID-19 virus; (2) is mandatory and applied indiscriminately to a general population; and (3) requires citizens to stay at home and refrain from or limit social and economic activities outside the home.

Three recent systematic reviews investigated the broader psychological impact of the COVID-19 pandemic on the general public worldwide. Luo, Guo, Yu, Jiang, and Wang (2020) revealed a pooled prevalence of anxiety and depression of 32 and 27%, respectively, among the general public. Similar results emerged in Salari et al. (2020), who found the pooled prevalence of stress, anxiety, and depression, is 29.6, 31.9, and 33.7% respectively. Vindegaard and Benros (2020) also found increased levels of depressive and anxiety symptoms along with general mental health symptoms. Based on their findings, Luo and colleagues concluded that ‘the COVID-19 pandemic has caused heavy psychological impact among medical workers and the general public’ (Luo et al., 2020, p. 7). Similarly, Salari and colleagues indicate that the COVID-19 pandemic ‘has not only raised concerns over general public health, but has also caused a number of psychological and mental disorders’ (Salari et al., 2020, pp. 8–11). Finally, Vindegaard and Benros suggest that ‘currently data is scarce, but indicates that mental health is affected in the general public’ (Vindegaard & Benros, 2020, p. 10).

These prior reviews raise important concerns regarding the mental health impact of COVID-19 lockdowns, but they are based on studies with significant methodological limitations, including cross-sectional designs and absence of control groups. As Meda and Slongo (2020) note, caution should be used when reporting conclusions on the psychological impact of the COVID-19 pandemic from cross-sectional studies lacking a proper control group. Given that prior reviews relied largely on cross-sectional studies, a review of more compelling evidence for the mental health effects of lockdown is needed. A stronger evidence base would have critical implications for policymaking and clinical interventions around the world.

The degree of media attention devoted to the psychological impact of COVID-19 may also create expectancy effects, consistent with the self-fulfilling prophecy (Merton, 1948) and the Pygmalion effect (Rosenthal & Jacobson, 1968). The capacity for resilience in response to the COVID-19 pandemic (i.e. a relatively stable trajectory of healthy psychological functioning) has sometimes been discounted, though it is the modal response to widely varying forms of acute adversity (e.g. Bonanno, Westphal, & Mancini, 2011; see also: Mancini, 2020). In sum, on the one hand, previous systematic reviews of cross-sectional research suggest that national lockdowns may have a heavy psychological impact. On the other hand, a resilience perspective suggests that the psychological impact of national lockdowns may be relatively small.

## Purpose of the present study

In the present study, we took advantage of a growing evidence base using more sophisticated methodologies. When experimental randomized trials are neither feasible nor ethical, both longitudinal within-person designs (with at least one data collection point before and one during the lockdown) and natural experiments involving a control group provide a methodologically more rigorous test of the effect of the COVID-19 lockdown. Because many lockdowns happened at a national level, natural experiments involving an appropriate control group were difficult to undertake. Nevertheless, longitudinal studies that examine within-person change before and after lockdowns provide important information on the psychological impact of lockdowns. The number of longitudinal studies and natural experiments assessing the effect of the COVID-19 lockdowns on mental health among the general population has multiplied during the last months. To provide a more rigorous assessment of the influence of the COVID-19 lockdowns, we conducted a review and meta-analysis of this evidence base to determine the psychological impact of COVID-19 lockdowns on the general population. We focused on broad dimensions of psychological functioning, including mental health symptoms, such as anxiety and depression, and positive psychological functioning such as well-being and life-satisfaction, consistent with the idea that these dimensions are separable but related constructs (Keyes, 2005). In addition, given the effect of lockdowns on in-person social interaction, we also examined feelings of loneliness and social support as ancillary outcomes.

## Method

### Knowledge synthesis

To synthesize the evidence in a timely manner, we chose a rapid review approach (e.g. Arksey & O'Malley, 2005; Khangura, Konnyu, Cushman, Grimshaw, & Moher, 2012; Tricco et al., 2015) over a systematic review methodology. Using guidance from Arksey and O'Malley (2005), we compiled a rapid review protocol (available from the corresponding author upon request).

### Information sources and literature search

To identify potentially relevant studies for inclusion, we adopted a search strategy that involved different sources: (1) electronic databases; (2) reference lists; (3) hand-searching of key journals; (4) existing networks; and (5) internet searches for unpublished papers. Specifically, searches were made on four electronic databases: Scopus, Web of Science, PubMed, and PsycInfo. We limited our search from January 2020 until June 2020. In addition, we undertook a gray literature search using Google Scholar. Finally, we consulted all citations of eligible articles and relevant review articles for supplementary references that were missed in the initial search (i.e. reference lists), hand-searched key journals to identify articles that have been missed in database (i.e. hand-searching of key journals), and we contacted experts in this field using existing knowledge and networks (i.e. existing networks).

Key search terms for mental health were these: adaptation, anxiety, depression, quality of life, mental health, mental illness, psychological symptoms, psychiatric symptoms, resilience, coping, stress, quality of life, well-being, distress, self-esteem, PTSD, loneliness, fear, social support, embeddedness, social cohesion, post-traumatic, post-traumatic, benefit findings, positive benefits, stress-related growth, and thriving. For the COVID-19 pandemic, the terms included COVID-19, coronavirus disease 2019, 2019-nCoV, novel coronavirus, SARS-CoV-2, quarantine, lockdown, and pandemic.

### Criteria for including studies in the review

#### Outcomes

We included for review primary human research studies that measured change or difference post-lockdown in at least one outcome related to mental health symptoms or positive psychological functioning. Mental health symptoms included assessments of depression, anxiety, posttraumatic stress disorder, suicidal ideation, negative affect, substance use, sleep disturbances, and general distress. Positive psychological functioning outcomes included assessments of satisfaction with life, positive affect, well-being, and quality of life. Scores on mental health symptoms and positive functioning were coded such as higher scores correspond to greater mental health symptoms and higher well-being, respectively. Social outcomes included loneliness and social support. In addition, we conducted subgroup analyses separately for each outcome when data were available from at least three studies.

#### Study design

We included studies that met the following criteria: (a) longitudinal designs assessing psychological functioning before and after COVID-19 lockdowns using the same instruments; (b) natural experiments comparing participants who were in lockdown with those who did not have such restrictions; (c) natural experiments with at least two (i.e. before and during the COVID-19 pandemic) cross-sectional data collection points (with different individuals) in which samples were matched or collected using the same methodology. We excluded retrospective studies and studies comparing the scores to norms or to assessments obtained from different studies. Our focus was on the general population, without age restriction, and, therefore, we excluded studies that focused on specific populations such as health care workers, survivors, or patient populations as well as studies investigating home self-quarantine. Guidelines, reviews, commentary, and non-English articles were also excluded. No studies were excluded based on sample size or study duration. Finally, we excluded studies with incomplete reporting of findings and statistics necessary for computation of effect sizes. For instance, we excluded studies using regression coefficients as meta-analytic input because this approach results in biased findings (Roth, Le, Oh, Van Iddekinge, & Bobko, 2018).

### Study selection

**Fig. 1.**
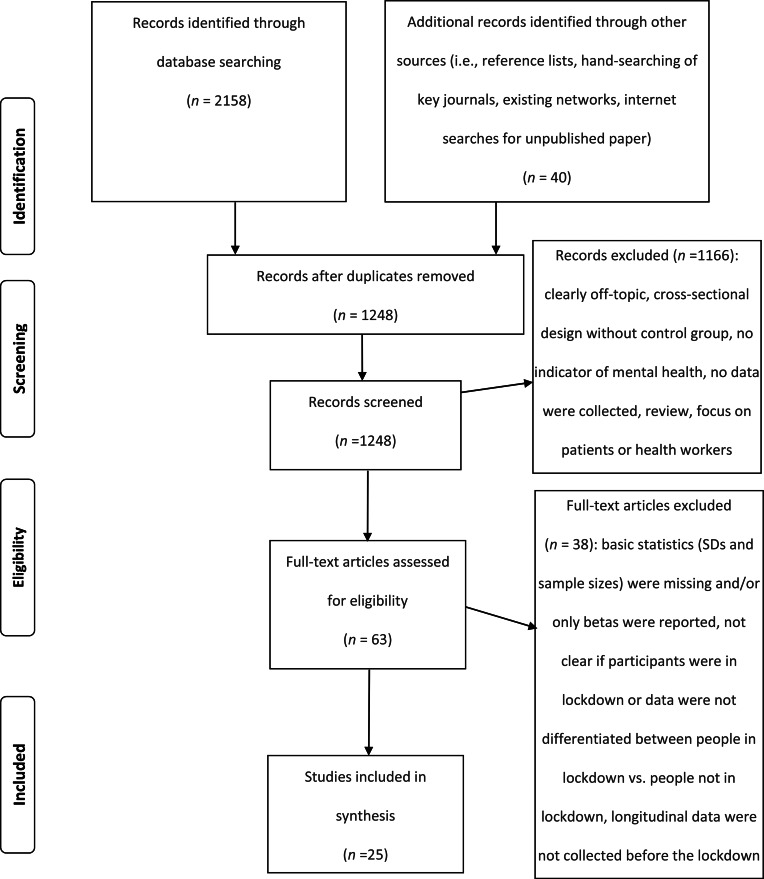
PRISMA flow diagram of the study selection process.

The literature search resulted in 2158 publications (Fig. 1). Moreover, we identified 40 publications through other sources (i.e. reference lists, hand-searching of key journals, existing networks, internet searches for unpublished paper). After removing duplicates, 1248 publications were screened for eligibility. Two reviewers (the authors of the present article) independently applied the inclusion and exclusion criteria to the records which were identified through the search. The reviewers screened for inclusion all publication titles and abstracts (*n* = 1248). The percentage of agreement between raters was high (98.7%). All disagreements were successfully resolved through discussion. The reviewers then independently assessed the full text of 63 publications for eligibility. There was no disagreement concerning the eligibility of studies identified for inclusion.

### Data items and data abstraction process

G.P. extracted data from the eligible studies into a customized Excel spreadsheet. The extracted data were independently verified by A.M. For each selected study, we recorded information as follows: first author, year of publication, study location(s), sources title, study populations (general population, adolescents, children, persons aged 60 or over), aims of the study, methodology, time passed since lockdown, peer-review status, participants' mean age, percentage of women, type of outcome measure, and main results. We conducted the following quality checks at each step: iterative consultation on data and any discrepancies, careful cross-checking of the data collected, and consensus decisions around methodology.

### Statistical analysis

We conducted a meta-analysis using the R *metafor* package version 2.4-0 (Viechtbauer, 2010). Only outcomes with data available from at least three studies were included. We calculated and transformed effect sizes to the bias-corrected Hedges' *g* following the guidelines of Borenstein, Hedges, Higgins, and Rothstein (2009). Values of 0.20, 0.50, and 0.80 were considered benchmarks for small, medium, and large effect sizes, respectively (Cohen, 1988). We conducted a random-effects meta-analysis using restricted maximum likelihood as a heterogeneity variance estimator (Langan, Higgins, & Simmonds, 2015). We assessed statistical heterogeneity using the *I*^2^ statistic which represents the percentage of total variation across effect size estimates that is due to heterogeneity rather than chance. According to Higgins, Thompson, Deeks, and Altman (2003), *I*^2^ values of 25, 50, and 75% are considered low, moderate, and high heterogeneity, respectively. To investigate publication bias, we used Begg's adjusted rank correlation test for funnel plot asymmetry (Begg & Mazumdar, 1994). In addition, we used a fail-safe *N* for effect size in meta-analysis (Orwin, 1983). This test quantifies the association between sample size and effect size, providing an estimate of the number of studies with null results that would be needed to reduce the average effect size to half the observed effect size.

We estimated effect size for different categories of mental health indicators. Effect size estimates for each category of outcomes are not statistically independent because each participant may be assessed using several different measures of outcome. To handle dependence among study effects, we conducted a full multivariate analysis of stochastically dependent effect sizes based on a linear random-effects model (assuming *ρ* = 0.7) using a robust variance estimation (Hedges, 2019).

To explain heterogeneity between studies we performed meta-regression analysis for outcomes with at least 20 effect size estimates. The following moderators were included: average age of participants, percentage of female participants, days passed since lockdown, peer-review status, study design, continent, and COVID-19 death rate. To determine COVID-19 death rate, we calculated COVID-19-related mortality per 1 000 000 population in the country or region of the study sample and at the midpoint of the time interval when data were collected (Johns Hopkins University Center for Systems Science and Engineering, 2020). We reported results from both univariate and multivariate meta-regression models. To assess the robustness of our meta-regression models, we conducted permutation tests (Higgins & Thompson, 2004) and reported permutation-based *p*-values and confidence intervals. Unless stated otherwise, we set *α* at 0.05 and all tests were two-sided.

## Results

We retrieved 2158 abstracts from the electronic databases and 40 additional records from other sources, of which 1248 remained after removing duplicates (Fig. 1). Following the screening of title and abstract, 63 articles were identified as potentially eligible studies. We assessed the full-text articles for eligibility, and 25 articles were included in the present meta-analysis because they fulfilled all eligibility criteria. This meta-analysis included 25 articles providing 58 effect size estimates. Of the 40 additional records from other sources, five were retained in the analysis. Online Supplementary Table S1 provides an overview of all included studies. Thirteen studies were conducted in Europe (Bojanowska, Kaczmarek, Kościelniak, & Urbańska, 2020; Daly & Robinson, 2020; Jackson, Garnett, Shahab, Oldham, & Brown, 2020; Kwong et al., 2020; Meda et al., 2020; Niedzwiedz et al., 2020; Ozamiz-Etxebarria, Dosil-Santamaria, Picaza-Gorrochategui, & Idoiaga-Mondragon, 2020; Pierce et al., 2020; Recchi et al., 2020; Schützwohl & Mergel, 2020; Shanahan et al., 2020; Stevenson, Wakefield, Drury, & Felsner, 2020; Zacher & Rudolph, 2020), six in Asia (Guo, Feng, Wang, & van Ijzendoorn, 2020; Lei et al., 2020; Li, Cao, Leung, & Mak, 2020; Liu et al., 2020; Wang & Zhao, 2020; Xin et al., 2020), five in North America (Bryan, Bryan, & Baker, 2020; Gratz et al., 2020; Luchetti et al., 2020; Tull et al., 2020; Zimmermann, Bledsoe, & Papa, 2020), and one in Oceania (Sibley et al., 2020). The majority of the included studies (*n* = 16) were peer-reviewed. Twelve studies used a within-person longitudinal design in which the participants were assessed before and after lockdown orders. Thirteen studies used a natural experiment design comparing regions or groups with and without lockdown orders. The number of days passed since lockdown varied from one to 60. All studies involved adult participants. The percentage of female participants ranged from 40 to 81%. Whereas 19 studies reported effects of lockdown on mental health functioning (e.g. depression, anxiety, general distress), six studies reported effects on positive psychological functioning (e.g. well-being, life satisfaction). Ten studies reported effect size estimates for depression and nine for anxiety. Seven studies provided effect size estimates for general distress. Five studies reported effect size estimates for social support. Three studies reported effect size estimates for loneliness and suicide risk.

### Meta-analysis

**Fig. 2.**
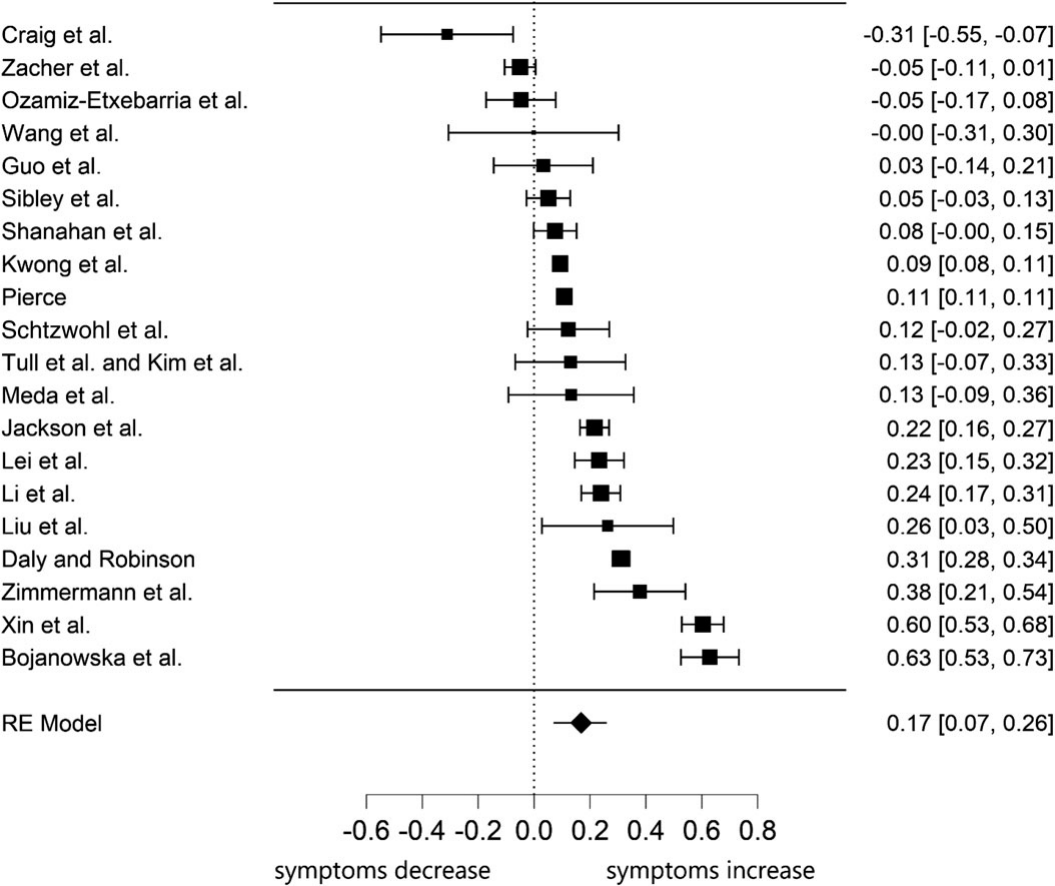
Forest plot of the 20 effect size estimates for the association between lockdown and mental health symptoms. *Note*. The year of publication was 2020 for all studies.

We calculated two summary estimates (estimated average effects), one for positive psychological functioning and another one for mental health symptoms (Table 1). Concerning mental health symptoms, the effect of lockdown was small and significant, *g* = 0.17, s.e. = 0.05, 95% CI (0.070.26), *p* < 0.001, with *I*^2^ = 99.05%, 95% CI (98.22–99.58), signifying large heterogeneity. Figure 2 depicts the forest plot on the impact of lockdown on effect size estimates for mental health symptoms. The Begg's adjusted rank correlation test for funnel plot asymmetry did not indicate the presence of publication bias, *τ* = −0.04, *p* = 0.823. Fail-safe *N* was equal to 19, indicating that 19 studies with non-significant results would have to be added to the meta-analysis to decrease the average effect size to half the observed effect size.

**Table 1.** Univariate analysis of effect sizes, heterogeneity, and fail-safe *N*

Variable	*k*	Effect size	s.e.	95% CI	*p*	*I*^2^ (%)	Fail-safe *N*
Mental health symptoms^a^	20	0.17	0.05	0.07–0.26	<0.001	99	19
Depression	10	0.15	0.07	0.01–0.30	0.037	94	10
Anxiety^b^	9	0.17	0.05	0.07–0.27	0.001	81	9
General distress^c^	7	0.12	0.08	−0.04 to 0.28	0.148	97	–
Positive functioning	6	−0.12	0.11	−0.33 to 0.09	0.274	99	–
Social support	5	0.03	0.02	−0.02 to 0.08	0.241	0	–
Loneliness	3	0.12	0.13	−0.13 to 0.37	0.336	72	–
Negative affect	3	0.25	0.20	−0.14 to 0.64	0.210	99	–
Suicidal ideation	3	0.14	0.33	−0.50 to 0.79	0.666	96	–

*Note*. *k* refers to the number of studies available for computation of a specific effect size (Hedges' *g*).

a Anxiety, depression, substance use, sleep disturbances, suicide risk, negative affect, and general distress symptoms.

b Anxiety and posttraumatic stress symptoms.

c Symptoms of general mental illness, perceived stress, psychological distress, and emotional distress. Higher scores on positive functioning correspond to higher well-being.

**Fig. 3.**
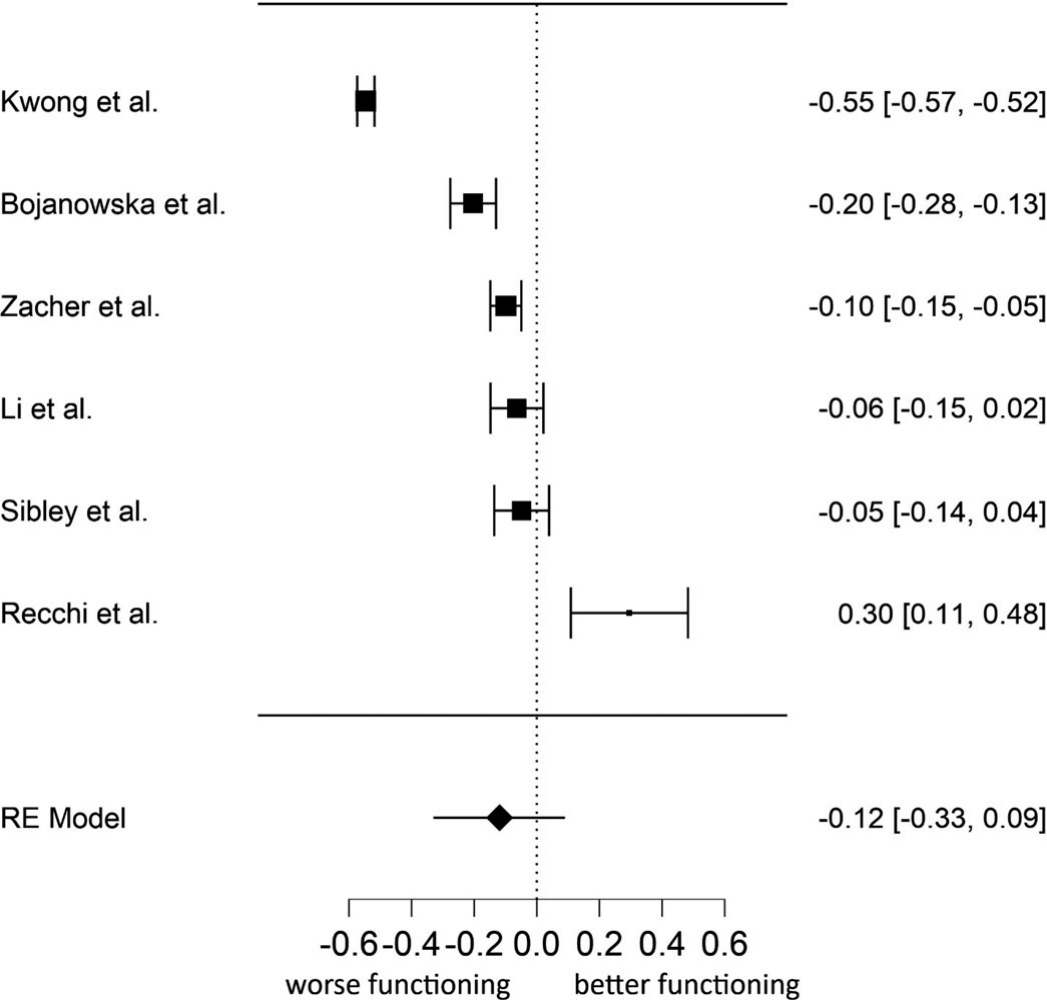
Forest plot of the six effect size estimates for the association between lockdown and positive functioning. *Note*. The year of publication was 2020 for all studies. Higher scores on positive functioning correspond to higher well-being.

The effect of lockdown on positive mental health was slightly smaller in magnitude than mental health symptoms and non-significant, *g* = −0.12, s.e. = 0.11, 95% CI (−0.33 to 0.09), *p* = 0.274, with *I*^2^ = 98.57%, 95% CI (96.19–99.78), indicating large heterogeneity. Figure 3 displays the forest plot on the impact of lockdown on effect size estimates for positive functioning. The Begg's adjusted rank correlation test for funnel plot asymmetry did not reveal evidence of publication bias, *τ* = −0.20, *p* = 0.719.

Next, we conducted a multivariate analysis of effect sizes. Using multivariate meta-analyses, it is possible to estimate the effect sizes for different dependent variables simultaneously in one model, while taking the relationship between the dependent variables into account. The results from basic central tendency statistics and publication bias are presented in Table 2. The results were very similar to the univariate analysis. The effect size estimates for anxiety and depression were quite small and significant, while those of social support, loneliness, distress, negative affect, and suicide risk were not significant. Except for social support, the effect size estimates showed very high heterogeneity. Table 3 displays results from full multivariate analysis of effect sizes based on a linear random-effects model. Consistent with univariate analysis, the only significant effect size estimates were those of anxiety and depression.

**Table 2.** Multivariate analysis of effect sizes based on random-effects model

Variable	Effect size	s.e.	95% CI	*p*
Anxiety	0.18	0.04	0.09–0.27	0.001
Depression	0.16	0.07	0.01–0.30	0.033
General distress	0.11	0.07	−0.03 to 0.25	0.107
Loneliness	0.07	0.09	−0.13 to 0.27	0.451
Negative affect	0.30	0.22	−0.16 to 0.76	0.187
Positive functioning	−0.16	0.09	−0.35 to 0.22	0.079
Social support	−0.01	0.04	−0.09 to 0.07	0.794
Suicide risk	0.13	0.23	−0.36 to 0.62	0.572

*Note:* Mental health symptoms were not included in the multivariate model because they were calculated using scores from anxiety, depression, substance use, sleep disturbances, suicide risk, negative affect, and general distress symptoms.

**Table 3.** Results of univariate and multivariate meta-regression models

Variable	Univariate				Multivariate			
	*β*	s.e.	95% CI	*p*	*β*	s.e.	95% CI	*p*
Study design	0.07	0.10	−0.12 to 0.26	0.467	0.10	0.16	−0.22 to 0.42	0.553
Publication status	−0.16	0.10	−0.35 to 0.03	0.973	−0.33	0.21	−0.74 to 0.08	0.160
Mean age	−0.00	0.00	−0.01 to 0.01	0.711	0.00	0.01	−0.01 to 0.01	0.983
Female participants (%)	0.00	0.00	−0.01 to 0.01	0.548	0.00	0.01	−0.02 to 0.01	0.658
Days in lockdown	0.01	0.00	−0.00 to 0.01	0.225	0.00	0.01	−0.02 to 0.02	0.883
COVID-19 death rate	0.00	0.00	−0.00 to 0.00	0.961	0.00	0.00	0.00–0.00	0.764
Asia	Reference				Reference			
Europe	−0.09	0.12	−0.31 to 0.15	0.459	−0.31	0.19	−0.68 to 0.06	0.132
North America	−0.16	0.17	−0.49 to 0.16	0.320	−0.32	0.21	0.16 to −0.73	0.164
Oceania	−0.19	0.24	−0.66 to 0.27	0.409	−0.18	0.31	−0.78 to 0.42	0.567

*Note*. Study design was coded as 1 = longitudinal, 0 = other study design; publication status was coded as 0 = non peer-reviewed, 1 = peer-reviewed. We reported results from permutation test on multivariate meta-regression model.

### Moderator analyses

Table 3 summarizes results of the univariate and multivariate meta-regression analyses for mental health symptoms. The omnibus test for the multivariate univariate model was not significant, *Q* = 6.73, *p* = 0.666. In both univariate and multivariate analysis, we found no effects for mean age, gender, continent, COVID-19 death rate, days of lockdown, publication status or study design.

## Discussion

The findings of our meta-analysis indicate a small but significant effect of COVID-19 lockdowns on mental health symptoms among the general population. Subgroup analyses indicated that depression and anxiety showed consistently small but significant effects of lockdown. However, we did not find evidence that lockdowns reduced positive psychological functioning, such as well-being, life satisfaction, or well-being. Furthermore, we did not find evidence that COVID-19 lockdowns increased loneliness or decreased perceptions of social support. Together these findings suggest that COVID-19 pandemic lockdowns had a selective and modest impact on mental health indicators but no effect on positive functioning (Keyes, 2005).

Although the point estimates indicate a small effect, this estimate should be interpreted cautiously given the relatively wide confidence intervals. Indeed, these findings should be understood in the context of substantial heterogeneity in the effect size estimates. Moderation analyses offered little to illuminate this heterogeneity, as days in lockdown, continent, publication status, COVID-19 death rate, sample composition, and study design were not associated with effect size. Heterogeneity between studies is likely due, in part, to the methodological challenges of studying the effects of lockdowns. These challenges included variation in research design, sampling strategies, mental health measures, and the availability of pre-pandemic assessments. Nevertheless, the heterogeneity in effect sizes is consistent with the wide degree of inter-individual variation in people's reactions to acute stress (Bonanno & Mancini, 2012). It is also consistent with the idea that the effects of lockdowns are not uniform and likely depend on a host of other contextual and person-centered factors.

Among the contextual factors that might have played an important role are citizens' attitudes toward lockdown measures. Indeed, there is evidence that positive attitudes toward lockdown measures predicted higher well-being and lower mental health symptoms during the COVID-19 lockdown in Italy (Prati, 2020). Polling data suggest that, in the early months of the pandemic, public approval of lockdown measures was high across countries YouGov (2020). Had attitudes toward lockdown been more negative, it is plausible that their effects on mental health would have been considerably more detrimental. However, this remains an open question and an important challenge for future research.

Most studies investigated the impact of COVID-19 lockdowns on anxiety and depression. In subgroup analyses, these were the only outcomes to produce significant effects. On the one hand, the inclusion of more studies may have allowed for effects to emerge for additional outcomes by increasing the statistical power of our tests. On the other hand, the effects were remarkably similar in magnitude across almost all outcomes, and we believe that if additional significant effects emerged because of increased power they would be likely to remain small.

In contrast to mental health outcomes, it is interesting to note that social support and loneliness were substantially unaffected by COVID-19 lockdowns. Although there have been concerns that the pandemic and the related containment measures would cause large increases in loneliness in scientific publications (e.g. Fiorillo & Gorwood, 2020; Killgore, Cloonan, Taylor, & Dailey, 2020), we did not find evidence supporting these predictions. It is plausible that containment measures such as lockdown, social distancing, and self-isolation, altered the ways in which people interacted but did not alter their perceived quality. Early in the pandemic, for example, a sharp increase in texting, social media, and video conference activity was observed (Richter, 2020). These technologies may have facilitated people's adaptation to the social restrictions imposed by lockdowns.

Another key point is that the widespread shared experience of the COVID-19 pandemic may have strengthened social cohesion and closeness because people may feel that ‘we are all in this together’ (Courtet, Olié, Debien, & Vaiva, 2020; Luchetti et al., 2020; Tull et al., 2020). Although there is some indication from repeated cross-sectional surveys that this may not be the case for COVID-19 (Borkowska & Laurence, 2020), there is prior evidence that disasters and pandemics can stimulate social cohesion and solidarity (e.g. Calo-Blanco, Kovářík, Mengel, & Romero, 2017; Hawdon & Ryan, 2011). After the severe acute respiratory syndrome epidemic in Hong Kong in 2003, for example, people reported increased feelings of embeddedness in the community and caring for friends and family members (Lau et al., 2008; Lau, Yang, Tsui, Pang, & Wing, 2006). The Fukushima nuclear disaster in 2011 also enhanced the importance of social connections (Uchida, Takahashi, & Kawahara, 2014). Indeed, as suggested by the ‘tend and befriend’ and ‘psychosocial gains from adversity’ theoretical perspectives, adversity can have favorable effects on affiliative behavior and potentially improve psychosocial functioning (Mancini, 2019; Mancini, Littleton, & Grills, 2016; Taylor, 2006). Despite the substantial effects of lockdowns on everyday life, the pandemic's capacity to enhance a sense of supportive others may have been a key factor in the small effect of the lockdown on mental health symptoms.

These potentially beneficial effects may also explain why we did not find evidence of significant increased suicide risk associated with lockdown (Joiner, Hollar, & Orden, 2006; Reger, Stanley, & Joiner, 2020). Indeed, there is evidence that social isolation and loneliness are associated with suicide risk (e.g. Calati et al., 2019; Van Orden et al., 2010). The experience of the COVID-19 pandemic may have changed the way people view health and mortality and make suicide less likely (Reger et al., 2020). As a result of the struggle with highly challenging life crises, people may change their views on mortality and experience positive psychological change including an increased appreciation for life (e.g. Tedeschi & Calhoun, 2004). However, we acknowledge that the present findings apply to the general population and that the impact of lockdown on suicide is likely to differ according to contextual and individual characteristics (John, Pirkis, Gunnell, Appleby, & Morrissey, 2020).

One way of understanding the small or non-significant effects of lockdown is people's innate capacity for psychological resilience. A considerable literature on widely varied stressful experiences has found that most people experience a stable pattern of adaptive functioning, or resilience, after an acute stressor (Bonanno, 2004). The present results remind us that – even after one of the most pervasive and restrictive interventions ever imposed on human beings – the average impact is small or non-significant (depending on the type of outcome), suggesting that most people retain their capacity for psychological resilience.

The present findings should not be taken as evidence that mental health problems do not occur in response to lockdowns. Moreover, the current findings only apply to the first lockdowns that were enforced during the early months of the COVID-19 pandemic. Given that the COVID-19 pandemic is likely to persist through 2021, the question of the psychological impact of repeated or prolonged lockdowns will remain open. Another important point is that small effects do not mean that the impact of COVID-19 pandemic lockdowns on mental health is trivial in applied terms. Even a very small effect size applied to the whole population could pose a significant public health problem. Moreover, this small effect size represents a mean value throughout the samples.

Meta-regression analysis failed to demonstrate that duration of lockdown moderated the effect of COVID-19 lockdowns on mental health. This finding does not mean that longer COVID-19 lockdown durations do not have any consequences for the mental health of the population. For instance, there are clear social and economic costs of lockdown policies (e.g. Miles, Stedman, & Heald, 2020), such as lost jobs and business closures which are thought to exert an important impact on mental health (e.g. Crayne, 2020). We contend that longer COVID-19 pandemic lockdowns policy *per se* may have a small and transient impact on population mental health as long as the social and economic consequences are limited. The findings that effect size estimates were not associated with both lockdown duration and death rates are in line with the results of a longitudinal study conducted in China during the pandemic (Wang et al., 2020). Although the number of COVID-19 deaths increased from the first to the second wave, no significant longitudinal changes in stress, anxiety and depression levels were found, and traumatic stress symptoms even decreased. Confidence in public health measures to control the spread of SARS-CoV-2 may help offset the psychological distress caused by lockdown. Finally, we point out that during a pandemic there are different stressors that may affect mental health, such as financial insecurity, perceived susceptibility to COVID-19 infections, and job stress. These stressors are not equally shared across countries, groups, and individuals. The findings of our meta-analysis cannot estimate or rule out the importance of other factors involved in a pandemic.

The meta-analytical findings should be interpreted with a number of limitations in mind. First, the small number of studies that investigated the outcomes of the present meta-analysis such as loneliness and suicide risk raise concerns about statistical power. We cannot rule out the possibility that non-significant effects are simply a power issue rather than a null finding. Due to the low number of studies investigating some of our outcomes, it is currently unclear whether our results can be generalized to these domains. A least five studies are needed to reasonably achieve adequate power from random-effects meta-analyses (Jackson & Turner, 2017), but it is worth noting that many of our analyses were based on five to 20 studies. Second, regarding participants' age, we were not able to find any studies involving children and adolescents and participants from different geographic areas such as Africa and South America. The psychological impact of lockdown on children may be different from that of adults, particularly in the context of widespread school closures. Therefore, the findings of our meta-analysis cannot be generalized to children and adolescents or to people all over the world. Indeed, while at a population level we found a small psychological impact of lockdown, we cannot rule out that specific subgroups would show different effects (e.g. children, care home residents, healthcare workers, people with preexisting mental health disorders, and people infected with COVID-19 virus). Third, the absence of a relationship between age and gender and effect size estimates should be interpreted with caution because the number of studies was not large. Fourth, moderator analyses of individual characteristics were limited to gender and age, because characteristics such as socioeconomic status, education, and working status were not reported in some studies. Fifth, another limitation of the review is that it does not tell us about the longer-term impact of lockdown on mental health. This is an especially important direction for future research, given that repeated or prolonged lockdowns were introduced to prevent the spread of the COVID-19 virus and that infections themselves may contribute to psychiatric disorders (Taquet, Luciano, Geddes, & Harrison, 2020). Sixth, in the current analysis, we did not take into account the stringency of the lockdown. We acknowledge that stringency of lockdowns varied somewhat, both across countries and across time. However, as we noted, all lockdowns share unique features, insofar as they restrict a wide variety of normative human behaviors. Future studies should focus on the psychological impact of different forms of lockdowns.

## Conclusion

The results of our meta-analysis indicate that the impact of COVID-19 pandemic lockdowns on mental health symptoms among the general population is small in magnitude. Therefore, claims that COVID-19 pandemic lockdown policies have a dramatic effect on population mental health are unsupported by the current findings. On the contrary, the findings suggest that people are largely psychologically resilient to stay-at-home orders, lockdowns, and similar restrictions that were enforced at the national or regional level around the world in response to the COVID-19 pandemic. Given the substantial degree of heterogeneity in our data, we posit that the impact of COVID-19 pandemic lockdowns on mental health may be different across different social groups and across different contexts and countries. Disparate health impacts have been an important focus during the COVID-19 pandemic and can be applied to the impact of lockdowns on mental health as well. Future research should attempt to establish a more precise relationship between lockdowns and positive and negative mental health indicators and to investigate the socio-contextual factors that are likely to influence such relationships. In conclusion, the effect size estimates of our meta-analysis of longitudinal studies and natural experiments represent the best available evidence on the effect of lockdown on mental health of the general population. Based on these estimates, the initial effect of lockdowns on mental health is relatively small, providing evidence of people's robust capacity for resilience.

## Supplementary material

For supplementary material accompanying this paper visit https://doi.org/10.1017/S0033291721000015. click here to view supplementary material
