# Supplementary material for: The psychological impact of COVID-19 pandemic lockdowns: a review and meta-analysis of longitudinal studies and natural experiments
Source: Psychol Med. 2021 Jan 13:1–11. doi: 10.1017/S0033291721000015 (PMC7844215; doi:10.1017/S0033291721000015)
Supplement: Supplementary file 1 [file S0033291721000015sup.zip › S0033291721000015sup001.docx]

Supplementary Table 1.

Overview of Studies Included in Meta-Analysis

| Author(s) | Title | Source title | Continent | Peer-reviewed | Longitudinal design | Average age | % female participants | Days passed since lockdown | Category of outcome |
| --- | --- | --- | --- | --- | --- | --- | --- | --- | --- |
| Bojanowska et al. | Values and well-being change amidst the COVID-19 pandemic in Poland | Pre-print | Europe | No | Yes | 54.66 | 55.00 | 28 | Satisfaction with life, positive and negative affect, eudaimonic well-being |
| Bryan et al. | Associations among state‐level physical distancing measures and suicidal thoughts and behaviors among U.S. adults during the early COVID‐19 pandemic | Suicide and Life-Threatening Behavior | North America | Yes | No | 45.20 | 50.70 | 1 | Depression and suicide ideation and attempt |
| Daly and Robinson | Problem drinking before and during the COVID-19 crisis in US and UK adults: Evidence from two population-based longitudinal studies | Pre-print | Europe | No | Yes | 51.30 | 53.70 | 34 | Drinking ≥ 4 times a week and heavy episodic drinking at least weekly |
| Guo et al. | Coping with COVID-19: exposure to covid-19 and negative impact on livelihood predict elevated mental health problems in Chinese adults | International Journal of Environmental Research and Public Health | Asia | Yes | No | 35.81 | 52.40 | 11 | Post-traumatic stress symptoms, depression, and insomnia |
| Jackson et al. | Association of the Covid-19 lockdown with smoking, drinking, and attempts to quit in England: An analysis of 2019-2020 data | Pre-print | Europe | No | No | 47.84 | 50.9 | 23 | High-risk drinking prevalence |
| Gratz et al. | Thwarted belongingness and perceived burdensomeness explain the associations of COVID‐19 social and economic consequences to suicide risk | Suicide and Life-Threatening Behavior | North America | Yes | No | 40.00 | 47.00 | 7 | Suicide risk |
| Kwong et al. | Mental health during the COVID-19 pandemic in two longitudinal UK population cohorts | Pre-print | Europe | No | Yes | 27.61 | 72.65 | 33 | Depression, anxiety, and well-being |
| Lei et al. | Comparison of prevalence and associated factors of anxiety and depression among people affected by versus people unaffected by quarantine during the COVID-9 epidemic in Southwestern China | Medical Science Monitor | Asia | Yes | No | 32.30 | 61.30 | 13 | Anxiety and depression |
| Li et al. | The psychological impacts of a covid-19 outbreak on college students in China: A longitudinal study | International Journal of Environmental Research and Public Health | Asia | Yes | Yes | 19.60 | 76.80 | 16 | Positive and negative affect and anxiety and depression |
| Liu et al. | Prevalence and predictors of PTSS during COVID-19 outbreak in China hardest-hit areas: Gender differences matter | Psychiatry Research | Asia | Yes | No | 35.00 | 54.40 | 10 | Posttraumatic stress symptoms |
| Luchetti et al. | The trajectory of loneliness in response to COVID-19 | American Psychologist | North America | Yes | Yes | 53.68 | 45.00 | 26 | Loneliness and social support |
| Meda et al. | COVID-19 and depressive symptoms in students before and during lockdown | Pre-print | Europe | No | Yes | 22.00 | 77.78 | 40 | Depression |
| Niedzwiedz et al. | Mental health and health behaviours before and during the COVID-19 lockdown: Longitudinal analyses of the UK Household Longitudinal Study. | Pre-print | Europe | No | Yes | 49.60 | 53.40 | 33 | Loneliness |
| Ozamiz-Etxebarria et al. | Stress, anxiety, and depression levels in the initial stage of the COVID-19 outbreak in a population sample in the northern Spain | Cadernos de Saude Publica | Europe | Yes | No | 32.94 | 81.10 | 1 | Depression, stress, and anxiety |
| Pierce et al. | Mental health before and during the COVID-19 pandemic: a longitudinal probability sample survey of the UK population | Lancet Psychiatry | Europe | Yes | Yes | 50.75 | 43.9 | 34 | Mental health symptoms |
| Recchi et al. | The “eye of the hurricane” paradox: An unexpected and unequal rise of well-being during the covid-19 lockdown in France | Research in Social Stratification and Mobility | Europe | Yes | No | 51.81 | 48.00 | 43 | Subjective well-being |
| Schützwohl and Mergel. | Social participation, inclusion and mental well-being following SARS-CoV-2 related lockdown restrictions in adults with and without mental disorders. Results from a follow-up study in Germany. | Pre-print | Europe | No | Yes | 40.30 | 75.50 | 28 | Mental health problems, depression, anxiety, and social support |
| Shanahan et al. | Emotional distress in young adults during the COVID-19 pandemic: evidence of risk and resilience from a longitudinal cohort study | Psychological Medicine | Europe | Yes | Yes | 22.00 | 48.10 | 25 | Perceived stress, internalizing symptoms, and anger |
| Sibley et al. | Effects of the COVID-19 pandemic and nationwide lockdown on trust, attitudes toward government, and well-being. | American Psychologist | Oceania | Yes | No | 51.70 | 66.60 | 9 | Distress, rumination, social support, and satisfaction with life |
| Stevenson et al. | Collectively coping with coronavirus: local community identification predicts giving support and lockdown adherence during the COVID-19 pandemic | Pre-print | Europe | No | Yes | 34.70 | 50.00 | 60 | Social support |
| Tull et al. | Psychological outcomes associated with stay-at-home orders and the perceived impact of COVID-19 on daily life | Psychiatry Research | North America | Yes | No | 40.00 | 47.00 | 7 | Depression, anxiety, loneliness, and social support |
| Wang et al. | The impact of COVID-19 on anxiety in Chinese university students | Frontiers in Psychology | Asia | Yes | No | 19.76 | 67.57 | 23 | Anxiety |
| Xin et al. | Negative cognitive and psychological correlates of mandatory quarantine during the initial COVID-19 outbreak in China | American Psychologist | Asia | Yes | No | 19.90 | 67.70 | 10 | Depression, self-harm or suicidal ideation, and emotional distress |
| Zacher and Rudolph | Individual differences and changes in subjective wellbeing during the early stages of the COVID-19 pandemic | American Psychologist | Europe | Yes | Yes | 44.70 | 39.90 | 10 | Life satisfaction and positive and negative affect |
| Zimmermann et al. | The impact of the COVID-19 pandemic on college student mental health: A longitudinal examination of risk and protective factors | Pre-print | North America | No | Yes | 18.40 | 76.20 | 19 | Depression and anxiety |

*Note*. The year of publication was 2020 for all studies.
