# Supplementary material for: The psychological impact of COVID-19 pandemic lockdowns: a review and meta-analysis of longitudinal studies and natural experiments
Source: Psychol Med. 2021 Jan 13:1–11. doi: 10.1017/S0033291721000015 (PMC7844215; doi:10.1017/S0033291721000015)
Supplement: Supplementary file 1 [file S0033291721000015sup.zip › S0033291721000015sup002.docx]

Searches were made on four electronic databases: Scopus, Web of Science, PubMed, and PsycInfo. We limited our search from December 2019 until June 2020. In addition, we undertaken a grey literature search using Google Scholar.

Search terms included the following:

"Adaptation, Psychological" OR "Anxiety" OR "Depression" OR "Quality of Life" OR “Mental health” OR “mental illness” OR “Psychological symptoms” OR “Psychiatric symptoms” OR “Resilience” OR “Coping” OR “Stress” OR “quality of life” OR “Well being” OR “Well-being” OR “Wellbeing” OR “Distress” OR “Self Esteem” OR “PTSD” OR “Loneliness” OR “fear” OR “social support” OR “embeddedness” OR “social cohesion” OR “post-traumatic” OR ““post-traumatic” OR “benefit findings” OR “positive benefits” OR “stress-related growth” OR “thriving”

AND

“COVID-19” OR “coronavirus disease 2019” OR “2019-nCoV” OR “novel coronavirus” OR “SARS-CoV-2” OR “Quarantine” OR “Lockdown” OR “Pandemic”
